# Decoding the molecular pathways governing trophoblast migration and placental development; a literature review

**DOI:** 10.3389/fendo.2024.1486608

**Published:** 2024-11-27

**Authors:** Lianlian Liu, Lin Tang, Shuai Chen, Lianwen Zheng, Xiaoyan Ma

**Affiliations:** ^1^ Department of Obstetrics and Gynecology, The Second Hospital of Jilin University, Changchun, China; ^2^ Obstetrics Department, Foshan Maternity and Child Health Care Hospital, Foshan, China; ^3^ Pathology Department, The Second Hospital of Jilin University, Changchun, China

**Keywords:** trophoblast migration, placental development, molecular pathways, Wnt signaling, preeclampsia, hypoxia-inducible factors

## Abstract

Placental development is a multifaceted process critical for a fruitful pregnancy, reinforced by a complex network of molecular pathways that synchronize trophoblast migration, differentiation, and overall placental function. This review provides an in-depth analysis of the key signaling pathways, such as Wnt, Notch, TGF-β, and VEGF, which play fundamental roles in trophoblast proliferation, invasion, and the complicated process of placental vascular development. For instance, the Wnt signaling pathway is essential to balance trophoblast stem cell proliferation and differentiation, while Notch signaling stimulates cell fate decisions and invasive behavior. TGF-β signaling plays a critical role in trophoblast invasion and differentiation, predominantly in response to the low oxygen environment of early pregnancy, regulated by hypoxia-inducible factors (HIFs). These factors promote trophoblast adaptation, ensure proper placental attachment and vascularization, and facilitate adequate fetal-maternal exchange. Further, we explore the epigenetic and post-transcriptional regulatory mechanisms that regulate trophoblast function, including DNA methylation and the contribution of non-coding RNAs, which contribute to the fine-tuning of gene expression during placental development. Dysregulation of these pathways is associated with severe pregnancy complications, such as preeclampsia, intrauterine growth restriction, and recurrent miscarriage, emphasizing the critical need for targeted therapeutic strategies. Finally, emerging technologies like trophoblast organoids, single-cell RNA sequencing, and placenta-on-chip models are discussed as innovative tools that hold promise for advancing our understanding of placental biology and developing novel interventions to improve pregnancy outcomes. This review emphasizes the importance of understanding these molecular mechanisms to better address placental dysfunctions and associated pregnancy disorders.

## 1 Introduction

### 1.1 Overview of placental development

Human placental development begins around the sixth to seventh day after conception (dpc) as the blastocyst attaches to the uterine wall (1, 2). Following adherence, the polar trophectoderm proliferates, resulting in the formation of a regional bilayer trophoblast comprising an inner layer of rapidly proliferating cytotrophoblasts (CTBs) and an outer layer of non-proliferating multinucleated syncytiotrophoblasts (STBs). Throughout pregnancy, both STBs and CTBs collaborate to sustain both placental structure and function (3). Placental villi development begins approximately eight days dpc, serving a crucial role in fetal nutrient transfer and maternal spiral arteries restructure. Trophoblast differentiation facilitates the invasive role of vascular remodeling and the establishment of maternal-fetal blood exchange that sustains gestation. This invasion requires interactions between endometrial decidual stromal cells (DSCs) and immune cells in the decidua (4).

During the eighth dpc, the placental villi commence development as the CTB proliferates. Around the twelfth dpc, the initial villous mesenchymal cores progress. At 15 dpc, endothelial cells of fetal origin can be found in the central part of the villi, forming cords that are recognized close to the basement membrane of the trophoblast. These findings revealed that trophoblast cells may contribute to the formation of the villous vascular network via paracrine signaling. Throughout the developmental stage between 18 and 20 dpc, the initial fetal capillaries and placental macrophages are found in the villous connective tissue. The capillaries of the placental villi continue to develop, and at approximately 22 dpc, vessels expand through endothelial cell cord connection and elongation in parallel to the villous axis. At 32 dpc, the placental blood vessels initiate a connection with the fetal circulation via the umbilical cord (5).

### 1.2 The role of the placenta in pregnancy maintenance

The placenta plays various vital biological roles during pregnancy, such as the transfer of nutrients and waste, gas exchange, passive immunity via the transfer of maternal immunoglobulins, and the secretion of hormones critical for fetal development (6). Due to the increased fetal metabolic demands, the placental and decidual arteries must undergo vascular remodeling, mainly facilitated by extravillous trophoblast cells (EVTs). EVTs remodel maternal spiral arteries, establishing low-resistance vessels to promote increased blood flow. Disturbing these processes can lead to complications, including preeclampsia (PE), highlighting the significance of the trophoblasts in vascular adaptations away from nutrient exchange (7).

The placenta can serve as a shield against infections and toxins, supported by expressing cytochrome P450 enzymes and immune mechanisms, including natural killer (NK) cells. The most prevalent decidual cells, DSCs, also play a crucial role in placental innate immune defenses (8).

The placenta secretes critical hormones, such as growth hormone, activins, and relaxin, which regulate maternal blood circulation and various metabolic processes (9). These hormones can affect the neuroendocrine organs that take part in the maternal processes, such as the brain and pituitary glands, helping in body adaptation to pregnancy and maintaining balance in the maternal body (9, 10).

Sex hormones, like estrogen and progesterone, have an essential impact through the pregnancy period by helping in the endometrial development of the cervix closure, in addition to uterine immune tolerance (11). Moreover, these hormones can affect the differentiation of trophoblasts and the regulation of placental steroidogenesis (12).

**Figure 1:**
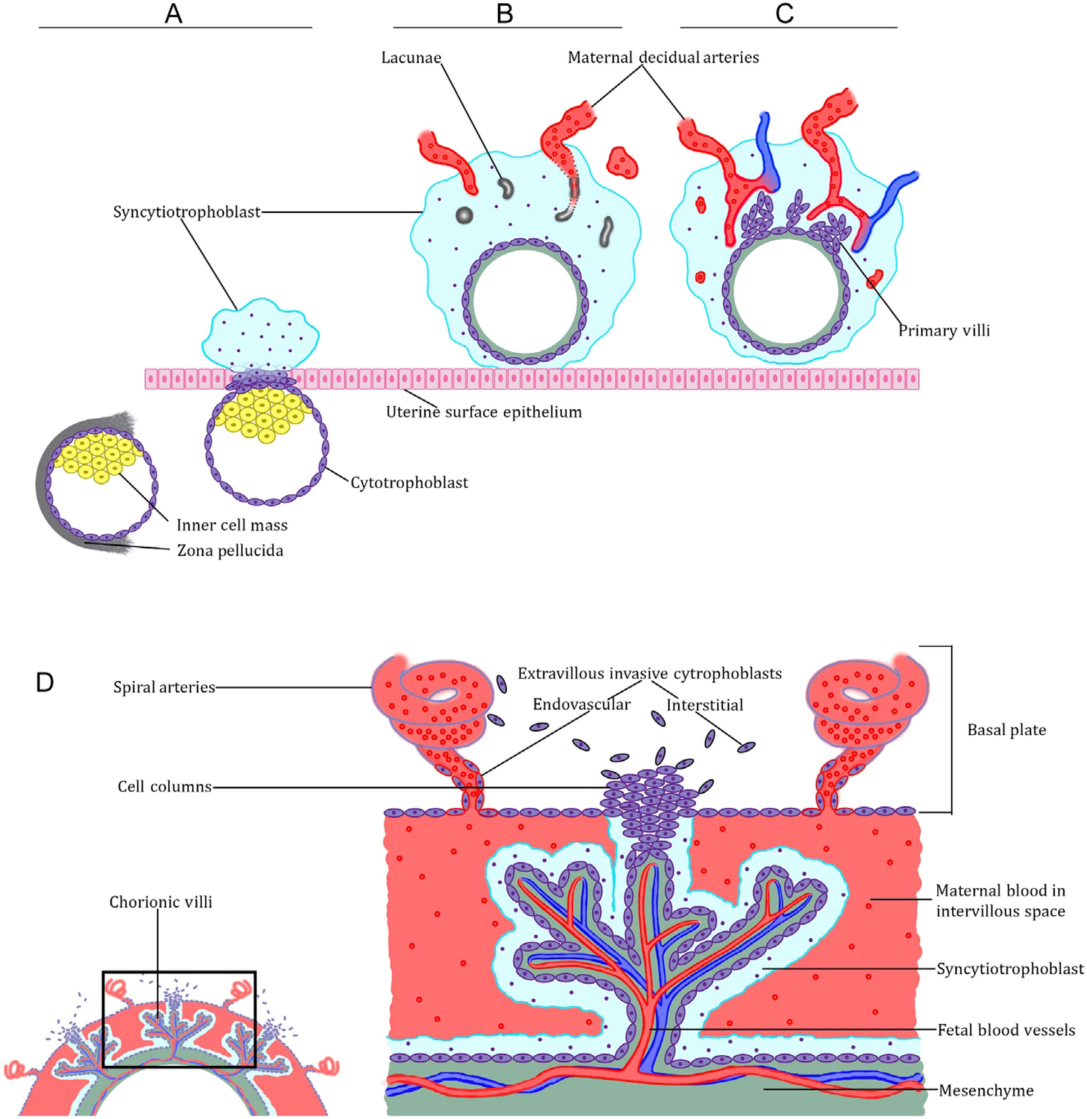
Overview of human placental development. **(A)** Preimplantation and peri-implantation blastocyst, showing both polar and mural trophectoderm, and the emergence of the primitive syncytiotrophoblast from the polar trophectoderm. **(B, C)** Postimplantation trophoblast development. Note the development of lacunae in the syncytiotrophoblast and the emergence of the primary villi. **(D)** The definitive placenta’s anatomy shows both the villous trophoblast organized into chorionic villi, and the extravillous cytotrophoblast cells organized into trophoblast cell columns (14).

A critical process in placental development is vascular remodeling, which is the reactive changes that occur in the walls of the uterine spiral arteries during human placental development. Upon encountering the extracellular matrix, the free CTBs undergo differentiation and transform into interstitial EVT (iEVTs). When iEVTs enter the arterial lumen, they transform into endovascular EVTs (enEVTs) (13). Subsequently, enEVTs deteriorate the media and smooth muscle and substitute the endothelium in the maternal arteries, creating high-capacity, low-resistance vessels to guarantee sufficient blood circulation to the placenta as the fetus develops and advances throughout pregnancy. **Figure 1** depicits a visual of human placental development. Therefore, the function of trophoblasts is essential in the process of restructuring blood vessels and contributes to the occurrence and progression of pregnancy disorders (15). Gaining insight into the functional regulation of the trophoblast is crucial for preventing and treating pregnancy disorders.

### 1.3 Understanding the trophoblast origin and functions

Trophoblasts perform a dual defense mechanism, providing a physical barrier and a chemical shield preventing the vertical transmission of different microorganisms. They produce type III antiviral interferons, which possess autocrine and paracrine mechanisms to limit infections. Additionally, placental extracellular vesicles (EVs) influence antiviral microRNAs that reveal a widespread antiviral activity (16).

Trophoblasts form the placenta and originate from the embryonic trophectoderm, which creates the first cell lining in the development of different mammals (17). Specific trophoblasts infiltrate the decidua in the hemochorial placental type, initiating direct contact with maternal blood (18, 19). Trophoblast stem cells (TSC) undergo rapid mitotic division and differentiate into special interstitial and endovascular trophoblasts, which demonstrate several migratory and invasive characteristics, enabling them to recognize and modify the behavior of other cell types at the point of connection between the maternal and fetal tissue.

Regulating cellular communication in this situation is tightly organized by factors released or expressed by trophoblastic cells in addition to other maternal factors. These factors may involve growth factors and interleukins. Trophoblast cells can break proteins such as collagen and fibronectin down in the extracellular matrix. This capacity enables cellular migration. Simultaneously, the decidua secretes various inhibitory proteins to regulate the migration of trophoblastic cells (20).

The successful invasion of trophoblasts is essential for remodeling uterine arteries and establishing sufficient blood flow for the developing fetus. Disturbances in the differentiation or invasion of the trophoblast can result in undesirable consequences, such as intrauterine growth restriction (IUGR) and PE (21–23). These complications are linked to the insufficient invasion of human trophoblasts and inadequate remodeling of the uteroplacental arteries (24, 25).

This review aims to conduct a comprehensive analysis of placental development and explore the cellular and molecular mechanisms and factors that play a role in coordinating the migration of trophoblasts within the uterus under both normal and abnormal conditions.

## 2 The main critical molecular pathways regulating trophoblast differentiation and proliferation

**Figure 2:**
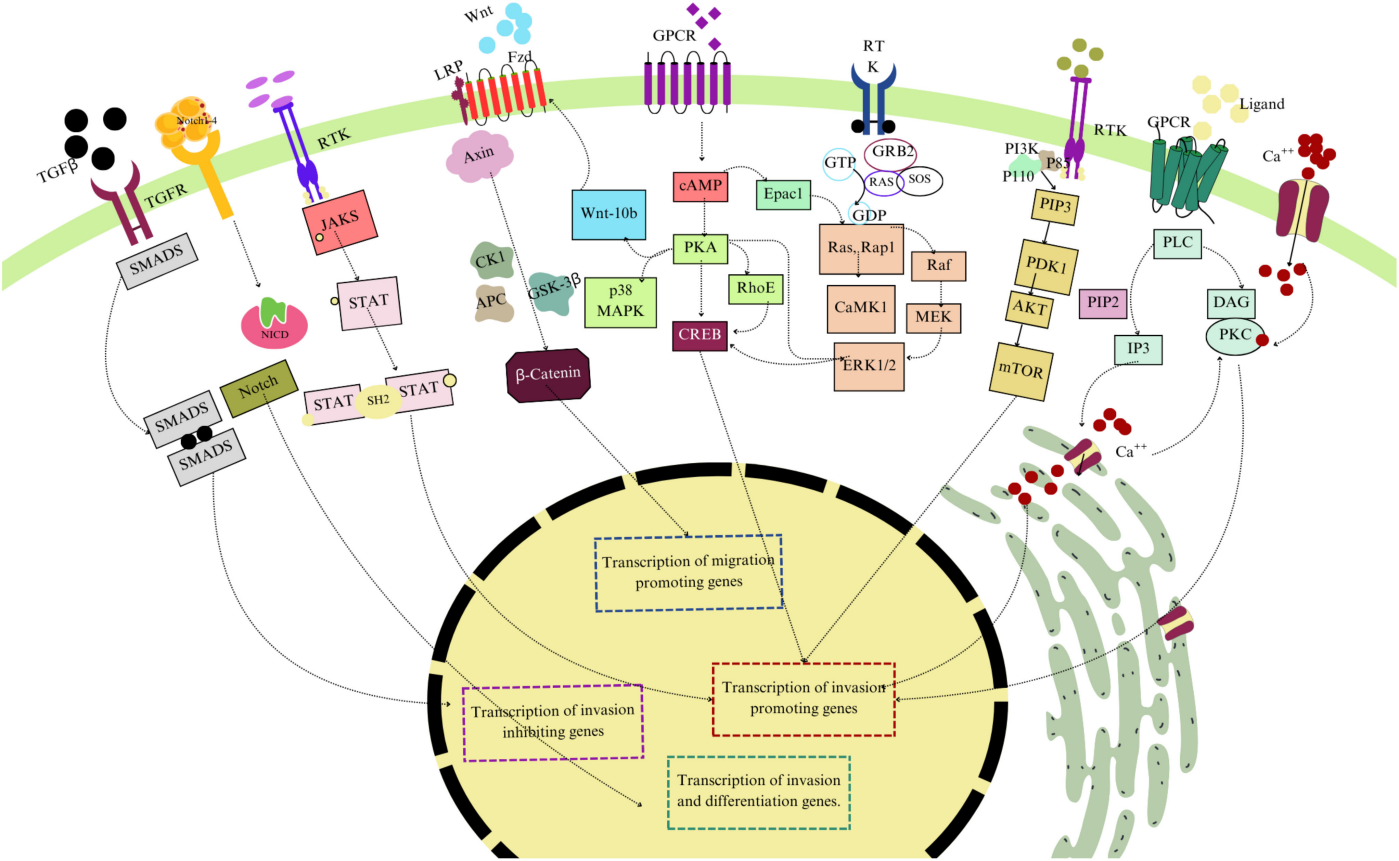
Overview of key molecular signaling pathways involved in the regulation of trophoblast migration and invasion. The diagram illustrates the TGF-β/SMAD pathway, Wnt/β-catenin pathway, JAK/STAT signaling, Notch Pathway, and PI3K/AKT/mTOR pathways. These pathways contribute to the transcriptional regulation of genes that either promote or inhibit trophoblast invasion and migration, essential for proper placental development and function. Different colors have been used to differentiate these factors.

Trophoblasts are the primary cellular elements of the placenta. Both trophoblasts’ development and function are regulated through several molecular signaling pathways. These pathways ensure trophoblast differentiation, proliferation, and invasion into maternal tissues, promoting typically developed placenta. This review explores critical pathways, highlighting their roles in trophoblast biology and their significance in placental disorders. **Figure 2** provides an overview of key molecular signaling pathways involved in the regulation of trophoblast migration and invasion.

### 2.1 The role of Wnt signaling in trophoblast differentiation and proliferation

Wingless ligands (Wnt) comprise a group of secreted proteins that significantly influence the homeostasis and development of various organs. This is achieved by maintaining equilibrium in cellular processes, including proliferation, invasion, differentiation, and cellular death (26). Studies showed that the dysregulation of the Wnt signaling pathway is linked to several pregnancy-related disorders, such as PE and IUGR (27, 28). Wnt signaling proteins have a vital impact on the gestation process by regulating the development of the placenta, determining the trophoblast lineage, facilitating the fusion of the chorioallantois, and contributing to the branching morphogenesis of the placenta (27).

Wnt proteins are a group of low-soluble glycoproteins. They are secreted in a form bound to lipoproteins or through exosomes (29). The initial member of the Wnt family ligands to be described was the Wnt1 proto-oncogene. In humans, researchers have identified nineteen Wnt ligands and ten Frizzled receptors (Fzd), which are 7-pass transmembrane proteins and members of the G protein-coupled receptor (GPCRs) superfamily (30). The Fzd proteins are recognized as the main receptors for Wnt ligands. These proteins bind to low-density lipoprotein receptor-related proteins (LRP) and form complexes that enable canonical and noncanonical Wnt signaling pathways (31).

#### 2.1.1 Canonical signaling pathway of Wnt

The canonical Wnt signaling is known mainly for stabilizing and moving β-catenin into the cell nucleus (32). In cells that are not being stimulated, β-catenin starts to degrade by the destruction complex, containing Axin and glycogen synthase kinase 3β (GSK-3β) proteins (33).

On the other hand, Wnt stimulation encourages creating a complex between Fzd and LRP to stabilize β-catenin in the cytoplasm. This disrupts the destruction complex of Axin, Adenomatous polyposis coli (APC), and GSK-3β. Disheveled (Dvl), a versatile protein, is brought to the cytosolic region of the receptor when Wnt binds to the cysteine-rich domain of Fzd, resulting in Axin and GSK-3β binding and LRP-5/6 phosphorylation by GSK-3β (34, 35). This event can either hinder the GSK-3β catalytic activity against β-catenin and promote its sequestration, or trigger the internalization and the subsequent degradation of the destruction of complex components. As a result, a rise in the levels of β-catenin in the cytoplasm occurs, and the de-phosphorylated β-catenin becomes active and moves into the nucleus, where it attaches to transcription factors that belong to the T-cell factor/lymphoid enhancer factor (TCF/LEF) family (35).

#### 2.1.2 Noncanonical signaling pathway of Wnt

Wnt proteins can autonomously influence different types of cells and can function away from β-catenin. The ligands of Wnt5a and Wnt11 can trigger the Wnt/PCP and Wnt/Ca^++^ signaling pathways by their attachment to Fzds and the independent stimulation of Dvl without the involvement of LRP, leading to activating phospholipase C, which enhances intracellular Ca^++^ concentration which ultimately triggers calcium/calmodulin-dependent kinase II, protein kinase C, and calcineurin (36, 37). The stimulation of Wnt5a leads to the accumulation of Ca^++^, which activates TGF-β-activated kinase (TAK1) and Nemo-like kinase. These kinases phosphorylate TCF, inhibiting its function and counteracting the effects of Wnt signaling (38). Therefore, in certain types of cancer, Wnt5a is a suppressor of tumor growth. However, the impact of Wnt5a is heavily influenced by the specific receptors it interacts with. Binding to Fzd2, Fzd3, Fzd5, and Fzd6 triggers Ca^++^ signaling. Yet the ligand has the potential to activate the canonical pathway when it interacts with Fzd4 and LRP (39). In addition, various Wnts have been shown to interact with the tyrosine kinases Ryk or Ror2, promoting the development processes without stimulating the traditional signaling pathways like ERK or PI3K/AKT/mTOR or Fzd involvement (40, 41). Overall, the binding of Wnts to the canonical or noncanonical receptors creates an intricate signaling network that exhibits significant overlap among the various pathways.

In **Table 1**, we have provided a comprehensive summary of both canonical and noncanonical Wnt signaling pathways and provided a summary of how they play their role in trophoblast development. We have highlighted the mechanisms, key components, and cellular impacts of each pathway, underlining the involvement of these pathways in crucial processes such as proliferation, differentiation, and invasion within trophoblast cells.

**Table 1:** A comprehensive overview of various Wnt signaling pathways, their mechanism, key components, cellular impact, and supporting references.

Pathway	Mechanism	Key components	Cellular impact	References
Canonical Wnt pathway	Stabilization of β-catenin, which translocates to the nucleus to act as a transcriptional co-activator.	β-catenin, Fzd receptors, Disheveled (Dvl), LRP5/6, GSK-3β, APC, Axin	Regulates cell cycle progression, differentiation, and invasion.	(32, 34)
Canonical pathway inhibition	β-catenin is phosphorylated by GSK-3β, leading to degradation through the ubiquitin-proteasome pathway.	GSK-3β, APC, Axin, β-catenin	Prevents β-catenin from initiating transcription, keeping the cell in a non-proliferative state	(33, 34)
Noncanonical Wnt/PCP pathway	Regulates cell polarity and movement by influencing cytoskeletal arrangements; does not involve β-catenin.	Wnt5a, Wnt11, Fzd receptors, Dvl, JNK, Rho, Rac, ROCK, Nemo-like kinase (NLK).	Impacts cell movement, polarity, and tissue architecture	(39, 40)
Noncanonical Wnt/Ca^2+^ pathway	Increases intracellular Ca^2+^ levels, triggering downstream kinases such as CaMKII and PKC.	Wnt5a, Fzd receptors, Dvl, PLC, IP3, Ca^2+^, PKC, Calmodulin-dependent kinase II, Calcineurin	Activates NF-κB and NFAT pathways; inhibits canonical Wnt signaling by preventing β-catenin transcription	(36, 42)
Wnt-dependent growth suppression	Wnt5a can suppress tumor growth by inhibiting canonical Wnt signaling, depending on receptor type.	Wnt5a, Fzd2, Fzd3, Fzd5, Fzd6	Suppresses cell proliferation and tumor growth through receptor-specific interactions.	(38)
Regulation by soluble inhibitors	Soluble inhibitors bind to Wnts and LRP to inhibit Wnt signaling.	Dickkopf (Dkk), secreted frizzled-related proteins (sFRPs)	Prevents canonical Wnt signaling by blocking interaction between Wnts and LRP5/6.	(43)
β-Catenin interaction with transcription factors	β-catenin can engage with transcription factors such as TCF, LEF, and others like steroid hormone receptors.	TCF, LEF, steroid hormone receptors	Regulates transcription of genes related to development and cellular processes, impacting multiple pathways.	(43)
Feedback regulation of Wnt components	Components like Axin and TCF are regulated through feedback loops upon Wnt pathway activation.	Axin, TCF	Modulates the duration and strength of Wnt signaling.	(35)
Role of transcriptional co-activators	Bcl9 and histone acetylases like p300 are activated to enhance transcription through the LEF-1/TCF complex.	Bcl9, histone acetylases (p300), LEF-1/TCF	Enhances the transcriptional response to canonical Wnt signaling, influencing cell differentiation and proliferation.	(35)

### 2.2 The impact of Notch signaling on trophoblast cell fate and function

The Notch family plays an essential role in evolutionary signaling, regulating a variety of cellular processes such as cell fate determination, cellular differentiation, invasion, and survival (44). In contrast to Wnt, the activation of Noth signaling necessitates direct interaction between cells through its ligands and receptors attached to the cell membrane (45). Four Notch receptors are present in mammals: Notch 1, 2, 3, and 4. They also possess five ligands: Delta-like (DLL) 1/3/4 and Jagged 1/2. Notch family proteins are present in the placenta, suggesting that Notch signaling may significantly affect the biological behaviors of trophoblasts (46). DLL4-Notch signaling regulates trophoblast action throughout early placental development, facilitating appropriate growth and vascular remodeling (47). During ligand binding, the Noth intracellular domain (NICD) undergoes cleavage. It translocates to the nucleus, where it interacts with the transcription factor recombination signal binding protein for immunoglobulin kappa J (RBPJκ) through its RAM (RBPJκ-associated module) domain for initiating the transcription of target genes, including members belonging to the Hairy/Enhancer of Split (HES) and Hairy/Enhancer of Split-related with YRPW motif (Hey) families (26). Hes and Hey proteins function as transcriptional repressors by interacting with Groucho/TLE proteins. This interaction inhibits gene expression, specifically genes involved in determining and differentiating cellular fate. Similar to the Wnt pathway, Notch signaling is characterized by a high level of complexity, primarily because of the multiple noncanonical effects of NICD and its interaction with most other pathways that regulate developmental processes. Various reciprocal regulatory interactions have been observed between Wnt and Notch, such as the phosphorylation of NICD by GSK-3 and the control of active β-catenin by membrane-bound Notch (48). It was found recently that DLL4-Notch signaling directly affects the migration and proliferation of trophoblasts, thereby regulating their function (49). The migration and proliferation of trophoblast is widely regarded as the pivotal biological process in early pregnancy. It has a key role in the proper placental development and ensures the provision of nutrition and oxygen to the fetus. The impaired migratory and invasive capacity of trophoblasts following embryo implantation can result in pathological complications during the later stages of pregnancy.

### 2.3 The impact of TGF-β/BMP pathway in regulating trophoblast proliferation and differentiation

Transforming growth factor-βs (TGF-βs) are a family of cytokines that include activins, inhibins, Nodal, and bone morphogenetic proteins (BMPs) (50). Studies have revealed the presence of TGF-β isoforms in the uterus during pregnancy in both human and murine models (51, 52); however, limited data exist regarding the precise impact of each isoform on trophoblast cells. Researchers highlighted that TGF-β may significantly impact the invasion of trophoblastic cells (53). The precise mechanisms by which it produces its effect remain inadequately investigated. Various murine models have yielded valuable knowledge regarding the molecular mechanisms involved in trophoblast proliferation, differentiation, migration, and invasion (54). The dysregulated TGF-β signaling has been linked to PE, caused by insufficient remodeling of spiral arteries and placenta dysfunction (55). As a result, this pathway is recognized to play a vital role in ensuring proper placentation.

Within the broader context of the TGF-β signaling pathway, BMPs represent a critical subset of at least 15 classic BMPs involved in various developmental processes (56). Ligand binding to this receptor complex in the canonical Smad-dependent pathway triggers the recruitment and phosphorylation of Smad 1, 5, and 9, which then form complexes with Smad4 and translocate to the nucleus to regulate target gene expression. In addition to the canonical Smad-dependent pathway, BMP signaling can activate noncanonical Smad-independent pathways involving TGF-β-activated kinase 1 (TAK1-p38), mitogen-activated protein kinase (MAPK), Cell division control protein 42 homolog (CDC42), Phosphoinositide 3-kinases (PI3-kinase), and LIM domain kinase (LIMK) (57). These pathways interact with other signaling networks, resulting in a complex and multifaceted downstream impact.

Graham and colleagues demonstrated the distinct impact of TGF-β in humans as it inhibits the development of extravillous trophoblast and enhances CTB syncytialization (58). It was reported that when villous from the human first-trimester placentas were exposed to TGF-β, the stimulatory effect caused by Activin was counteracted (59). Conversely, thymine-guanine-interacting factor 1 (TGIF-1), a protein that inhibits TGF-β, is noticeably higher in mature syncytiotrophoblasts than immature cytotrophoblasts in human placentas during the first and third pregnancy trimesters. Reduced expression of *TGIF-1* is linked to diminished differentiation of syncytiotrophoblasts, as proved earlier (60).

The distinct functions of Smad2 and Smad3 within the TGF-β signaling pathway have garnered attention, particularly in relation to their roles in trophoblast differentiation and invasion. Smad2 and Smad3, although structurally comparable and participating in TGF-β signaling, demonstrate different functions during gestational stages (61). Total Smad2/3 levels remained relatively stable throughout gestation; however, the ratio of phosphorylated (active) to total Smad2/3 fluctuated across gestational stages, with a higher phosphorylated/total Smad2 ratio observed in late gestation and a higher phosphorylated/total Smad3 ratio in early gestation (62).

The *Smad2* knockdown enhanced trophoblast invasion, upregulated the levels of endovascular trophoblasts (enEVT) markers mRNA, and augmented the capacity of trophoblasts to establish endothelial-like networks; however, the contrary effect was observed with Smad3 knockdown (63). Smad2 siRNA enhanced the EVT proliferation in early gestation placental specimens, though Smad3 siRNA diminished the EVT proliferation (64). These reports highlighted that Smad2 and Smad3 exhibit opposing roles in the development of an enEVT-like phenotype. Smad2 can suppress extravillous trophoblast differentiation and invasion, while Smad3 can promote them.

### 2.4 The role of vascular endothelial growth factor in placental vascular development

Vascular endothelial growth factor (VEGF) is crucial in promoting angiogenesis and vascular permeability. It plays an essential role in various processes during pregnancy, such as trophoblast proliferation and migration (65). A recent study has highlighted the significance of VEGF genetic polymorphism as a genetic predictor for PE (66). The VEGF family consists of several proteins, such as placental growth factor (PlGF), endocrine gland-derived vascular endothelial growth factor (EG-VEGF), and VEGF-A to VEGF-F (67). VEGF-A (can be called VEGF) was initially documented in 1983 (68). The proteins fms-like tyrosine kinase 1 (Flt-1, or VEGFR-1), kinase insert domain receptor (KDR, or VEGFR-2), and Flt-4 (VEGFR-3) are referred to as VEGFRs (67).

VEGFR-2, which has the most potent pro-angiogenic effects, exhibits more significant tyrosine kinase activity than VEGFR-1. VEGFR-1 has more capability to interact with VEGF, VEGF-B, and PlGF with inhibited kinase activity, so it acts as a negative VEGF regulator (65). Consequently, the soluble VEGFR-1 form, sVEGFR-1, can capture VEGF, VEGF-B, and PlGF, preventing them from binding to membrane receptors. This action has been shown to have a significant association with recurrent miscarriage, adverse pregnancy outcomes, and unexplained infertility.

Placental trophoblasts also produce sFlt1, which is crucial in regulating the VEGF signal from the maternal endometrium (69, 70). Overproduction of sFlt1 during pregnancy in humans is linked to severe pregnancy complications (70, 71). Therefore, comprehending the significance of VEGF signaling in pregnancy has implications not only for understanding placental development but also for understanding the causes of pregnancy complications.

#### 2.4.1 The adaptation of trophoblasts to low oxygen environments in early pregnancy through the hypoxia-inducible factor

During the initial stages of pregnancy, trophoblasts experience a hypoxic microenvironment, which is crucial for the TSC to maintain a stable internal environment, protect DNA damage, and differentiate (72, 73). Several studies highlight the importance of the hypoxic environment for the blastocyst to attach to the uterus, initiating placental proliferation through the invasive trophoblast cells and maintaining the stem cell state of trophoblasts (74, 75).

Hypoxia-inducible factor (HIF) can influence numerous cellular processes reacting to low oxygen levels, including angiogenesis, migration/invasion, erythropoiesis, and cell metabolism (76). HIF is a protein comprising two subunits: an alpha subunit with two forms (HIF-1α and HIF-2α) and a beta subunit (HIF-1β, also known as Arnt). Although HIF-1β is not affected by O_2_, HIF-1α undergoes rapid degradation in the presence of O_2_. HIF-1α is present in significant quantities during the 7–9-week period of gestation when the oxygen level in the placental microenvironment is about 2%. However, its levels decrease during the 10–12-week gestation period when oxygen levels rise to about 8% (77). A study conducted with *HIF1* knockout mice revealed that the absence of either the HIF-1α or HIF-1β subunit in fetal trophoblasts leads to abnormal placental development. This is characterized by an inadequate fusion of the chorion and allantois and a reduction in the spongiotrophoblast and labyrinth layer (78).

The maternal HIF-1α is necessary for placentation by attracting uterine NK cells and trophoblasts to the maternal decidua and influencing trophoblasts’ function (78). Insufficient expression of HIF-1α in the placental maternal or fetal side may be linked to impaired vascular development, comparable to the vascular development issues observed in PE (73).

### 2.5 The ERK pathway in the migration and invasion of trophoblast

The fundamental role of the extracellular signal-regulated kinase (ERK) pathway remains in the regulation of cell proliferation, differentiation, and survival as a part of the broader MAPK signaling cascade (79). It is typically activated through receptor tyrosine kinases (RTKs) in response to extracellular signals, such as growth factors. When activated, the ERK pathway plays a role in the phosphorylation of downstream kinases, eventually activating transcription factors like ETS Like-1 protein Elk-1 (ELK1) and Activator protein 1 (AP-1). These transcription factors initiate the expression of several genes involved in cell moment, proliferation, and invasion (80).

The trophoblast cells should go through migration and invasion; integral processes for successful implantation and placental development remain dependent on the activation of the ERK pathway (81). Experimental studies have demonstrated that inhibiting ERK signaling can reduce trophoblast invasiveness, indicating that proper ERK activity is essential for these cells to invade maternal tissues and form the placenta. Since dysregulated ERK signaling is often associated with cancer cell metastasis, its role in trophoblasts emphasizes a similar mechanism of controlled invasiveness during placental formation (82).

### 2.6 JAK/STAT signaling pathway in trophoblast differentiation and function

The Janus kinase/signal transducer and activator of transcription (JAK/STAT) signaling pathway is activated in response to cytokines and growth factors, making it a critical regulator of cell proliferation, differentiation, and immune responses (83). In trophoblasts, the JAK/STAT pathway mediates various aspects of cell function, including differentiation and invasion. For instance, STAT3 activation has been shown to promote the differentiation of cytotrophoblasts into more invasive EVTs, a process essential for proper placental attachment and remodeling of maternal spiral arteries (55, 84).

The pathway begins when extracellular cytokines, such as interleukins, bind to their respective receptors, activating JAK kinases. Activated JAKs phosphorylate STAT proteins, which then dimerize and translocate to the nucleus to initiate transcription of target genes (85, 86). Dysregulation of the JAK/STAT pathway has been linked to pregnancy disorders, such as PE, which are characterized by inadequate trophoblast invasion and improper placental formation (87). These findings highlight the essential role of JAK/STAT signaling in maintaining a healthy pregnancy by regulating trophoblast behavior.

### 2.7 PI3K/AKT/mTOR pathway in regulating trophoblast growth and survival

The phosphatidylinositol 3-kinase (PI3K)/AKT/mammalian target of rapamycin (mTOR) pathway is a central signaling network that governs cell growth, metabolism, and survival (88). In trophoblast cells, this pathway is activated by various signals, including growth factors, hormones, and integrins, which bind to RTKs or GPCRs. Once activated, PI3K catalyzes the production of phosphatidylinositol (3–5)-trisphosphate (PIP3), which recruits and activates AKT. Activated AKT can phosphorylate numerous downstream targets, including mTOR, to regulate cellular processes such as protein synthesis, glucose metabolism, and cell survival (89).

For trophoblasts, the PI3K/AKT/mTOR pathway is crucial in promoting cell proliferation, migration, and invasion—key processes during placental development. This pathway also contributes to the cells’ ability to adapt to stress, such as low oxygen levels, which are prevalent during early pregnancy (90, 91). Crosstalk between the PI3K/AKT/mTOR pathway and other pathways, such as Wnt and TGF-β, ensures a coordinated response to various signals, allowing trophoblasts to differentiate and invade maternal tissues effectively (92, 93). Dysregulation of this pathway has been associated with conditions such as intrauterine growth restriction (IUGR) and PE, underscoring its importance in placental health.

## 3 Regulatory mechanisms impacting trophoblast differentiation and function

Trophoblast differentiation is a strictly regulated process controlled by several mechanisms. These mechanisms include epigenetic regulation, non-coding RNAs, and essential transcription factors. All these factors orchestrate the maintenance of proper placental development. Dysregulation in these mechanisms can lead to several pregnancy complications.

### 3.1 Impact of epigenetic regulation and DNA methylation

DNA methylation is crucial for the preservation of stem cells and the facilitation of lineage commitment. Specifically, DNA methylation plays a role in controlling stem cell aging, renewal, and specialization (94). The process of human trophoblast differentiation is accompanied by significant alterations in gene expression (95). The impact of DNA methylation on these transcriptional pathways remains inadequately clarified. Several studies have either focused on a limited number of genes or provided a broad overview of methylation data (95–97). Until recently, the lack of sufficient studies on human trophoblast has hindered our comprehension of the effects of DNA methylation on early trophoblast differentiation events in humans (98, 99).

Vlahovic and colleagues demonstrated the significance of DNA methylation in the development of the placenta, as suppressing methylation in pregnant rats leads to smaller placentae with structural abnormalities and modified proportions of trophoblast populations (100). Branco and colleagues demonstrated similar findings in knockout models of DNA methyltransferases, with embryonic lethality occurring due to impaired trophoblast differentiation (101). The influence of DNA methylation on trophoblast development initiates around 5 dpc after fertilization (102). The separation of murine TSC into embryonic and extraembryonic tissue is linked to distinct methylation patterns in important genes such as caudal type homeobox 2 (*Cdx2*), eomesodermin (*Eomes*), E74 like ETS transcription factor 5 (*Elf5*), placenta expressed transcript 1 (*Plet1*), and transcription factor AP-2, gamma (*Tcfap2c*). These changes in methylation can be associated with differences in gene expression between these lineages (102, 103).

Although the extent to which DNA methylation controls the process of murine trophoblast differentiation has not been thoroughly investigated, it is evident that specific genes, such as *Elf5* promoter hypomethylation, exhibit comparable levels of methylation across trophoblast subsets. There is also distinct gene methylation variation, such as in the enhancer region of dimethylarginine dimethylaminohydrolase 2 (*Ddah2*), among different murine trophoblast lineages (104, 105). Additionally, The TSC state and TSC self-renewal are established in mice through the hypomethylation of *Elf5* and *Plet1* promoters. The impact of similar DNA methylation events on human TSC function and differentiation remains unclear. However, previous research has indicated a variation in methylation levels between the promoter regions of *ELF5* in human CTBs and EVTs (106).

Although murine and human trophoblasts share certain transcriptional and regulatory mechanisms in lineage segregation, there are distinct anatomical differences in their early differentiation pathways. Furthermore, the mechanisms that ensure the conservation of methylation networks across species are not fully understood.

### 3.2 The role of microRNAs and non-coding RNAs in post-transcriptional regulation of trophoblast functions

The non-coding transcripts can be categorized into two main groups based on their size: non-coding RNAs (ncRNAs) that have 200 or fewer nucleotides, which are referred to as small RNAs, and non-coding RNAs that exceed 200 nucleotides, known as long non-coding RNAs (lncRNAs) (107). However, lncRNAs, which are the most diverse and extensive group of ncRNAs, play a role in various biological processes, including imprinting, cell cycle control, nucleus-cytoplasm transport, nuclear architecture, transcriptional and post-transcriptional regulation, and epigenetic regulation as mentioned previously (108–110).

In the embryonic development, microRNAs (miRNAs) regulate several stages. Circulating miRNAs such as miR-187-5p, miR-204-5p, miR-449a, and miR-519a-3p in the peripheral maternal blood mainly originate from trophoblasts (111, 112). Trophoblasts exhibit the expression of specific clusters of microRNAs: the miR-371-3 clusters, chromosome 14 miRNA clusters (C14MC), and chromosome 19 miRNA clusters (C19MC) (113). These clusters of miRNAs are demonstrated to be associated with the formation of the placenta (114). The expression of miRNAs in C14MC gradually declined throughout pregnancy, whereas the expression of C19MC and members of the miR-371-3 cluster notably increased (115). Consequently, it can be predicted that miRNAs could potentially function as serum markers linked to human pregnancy.

Furthermore, a growing body of research indicates that the varying levels of different circulating miRNAs during pregnancy are strongly linked to the onset and progression of pregnancy disorders (116, 117). The expression profile of circulating miRNA in complicated pregnancies differs from that of normal pregnant women. These differences include the dysregulation of miRNA: miR-125a-5p, miR-125b, miR-182-5p, miR-210, miR-218-5p, miR-320a, miR-525-5p (118–121). This evidence highlights that miRNAs found in the maternal bloodstream have the capacity to offer novel diagnostic and therapeutic opportunities for pregnancy complications. In addition, miRNA cluster members regulate various trophoblast cellular functions, including invasion, proliferation, migration, differentiation, and apoptosis (122, 123).

LncRNAs also regulate trophoblast role by maintaining gene expression. The expression of the lncRNA, Small nucleolar RNA hostgene 7 (SNHG7) is decreased in the villi of patients with recurrent spontaneous abortion (RSA). Moreover, Jiang and colleagues demonstrated the enhanced SNHG29 expression in the placentas of preterm birth cases (124). Zhu and colleagues demonstrated the upregulation of the trophoblastic lncRNA, Maternally Expressed Gene 8 (MEG8) levels in RSA, which inhibited proliferation and invasion (125). Another study highlighted that reduced lncRNA plasmacytoma variant translocation 1 (PVT1) placental levels in gestational diabetes mellitus (GDM) and PE patients compared to healthy placenta hindered trophoblastic invasion and proliferation (126).

### 3.3 Key transcription factors regulating trophoblast lineage developmental process and specific functions

Studies with murine models have demonstrated that specific transcription factors and cofactors control the complex trophoblast cell lineage development process. These factors regulate gene expression during different stages, including the development of trophoblast cells in the early embryo, the growth of TSCs after embryo implantation and at the beginning of placenta formation, and the differentiation of trophoblast progenitors into specialized trophoblast subtypes in the proliferative and mature placenta.

The presence of important trophoblast stem state factors, including transcription factor AP-2 gamma (TFAP2C), GATA-binding proteins 2 and 3 (GATA2/3), and E74-like ETS transcription factor 5 (ELF5), is observed in both human CTBs and mouse TSPCs (127). This suggests that these shared factors may play a role in establishing similar regulatory programs in humans and murine models, controlling the balance between stemness and differentiation.

TEA domain transcription factor 4 (TEAD4) a transcription factor belonging to the TEA domain family, has been recognized as a crucial upstream regulator in trophectoderm (TE) development. Home and colleagues demonstrated that the expression of TEAD4 is conserved in the TE lineages of various human and murine blastocysts (128). Interestingly, Tead4 mRNA expression has been observed in the TSPCs of a postimplantation mouse embryo and the TE lineages found in preimplantation embryos (129). The expression of TEAD4 is preserved in the progenitors of CTB in a human placenta through the first trimester. Saha and colleagues discovered that TEAD4 plays an essential role in preserving the undifferentiated state and stimulating the ability of trophoblast progenitor cells to regenerate themselves in embryos after implantation. This is vital for the successful development of the placenta and the advancement of pregnancy.

Genes specific to trophoblast cells, such as *Cdx2*, *GATA3*, and *Fgfr2*, do not increase their expression in TEAD4 knockout mice, leading to the death of the embryo before implantation. The Hippo pathway rigorously regulates the activity of TEAD4. During the initial stage of mouse blastocyst development, the Hippo signaling pathway is not active in the outer layer of cells that will eventually form the TE. As a result, the Yes-associated protein 1 (YAP), produced by the *Yap1*, exists in an unphosphorylated state. YAP, which is not phosphorylated, is found in the nucleus and forms a partnership with TEAD4 to activate TE specifiers such as Cdx2 and GATA3 (130).

During the initial stage of development, specifically in the inner cell mass (ICM), the Hippo pathway becomes active and triggers the phosphorylation of YAP. This phosphorylation process promotes the retention of YAP in the cytoplasm and its subsequent degradation through the proteasome. The transcriptional coactivator with PDZ binding motif (TAZ), encoded by the *Wwtr1*, has a functional role similar to YAP’s in the early mouse embryo (131). Recent studies have discovered significant differences in the regulation of the Hippo pathway during early development between mice and humans (132, 133). In addition, YAP activity enhances the generation and self-renewal of human naïve pluripotent stem cells.

Moreover, it has been shown that idiopathic recurrent pregnancy failures are linked to a decrease in the expression of TEAD4 in trophoblast progenitors (129). By depleting YAP1, it was discovered that it promotes self-renewal, development, and proliferation by regulating genes related to the cell cycle and STB (134).

**Figure 3:**
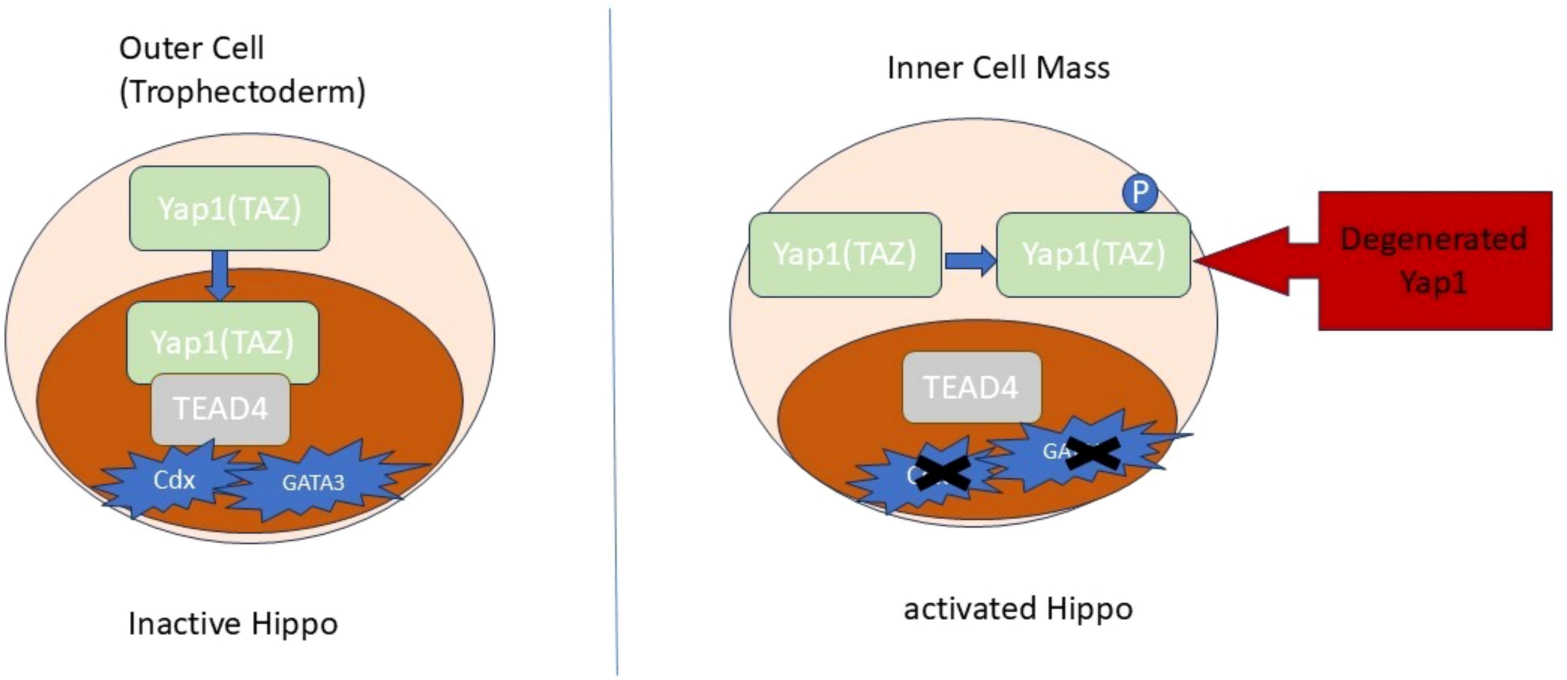
The Hippo signaling in trophoblast differentiation. The translocation of Yap1 to the nuclei is responsible for determining the specification of TE at the 8-16-cell stage. The suppression of the Hippo pathway facilitates the movement of Yap1/TAZ proteins into the nuclei, where they engage with TEAD4 to stimulate the expression of specific genes.

Additionally, GATA3/2 and TFAP2C showed an elevated expression in TE and CTB (135), controlling the development of trophoblast cells and aiding in the transition from pluripotency. The crucial functions of GATA2/3 and TFAP2C in the human CTB are anticipated, as their absence in the murine system hindered trophoblast development, ultimately causing the death of the embryo (136). **Figure 3** provides a visual summary of the hippo signaling in trophoblast differentiation.

The DNA methylation-mediated regulation of the Elf5/ELF5 promoter is conserved in both species (137, 138). ELF5 in mice works with EOMES and TFAP2C to function as a molecular switch that controls the equilibrium between the proliferation and differentiation of TSCs (105). ELF5 plays a crucial role in the development of the placenta in mice and the maintenance of TSCs. However, its specific function in the human trophoblast has not yet been fully understood (139).

**Figure 4:**
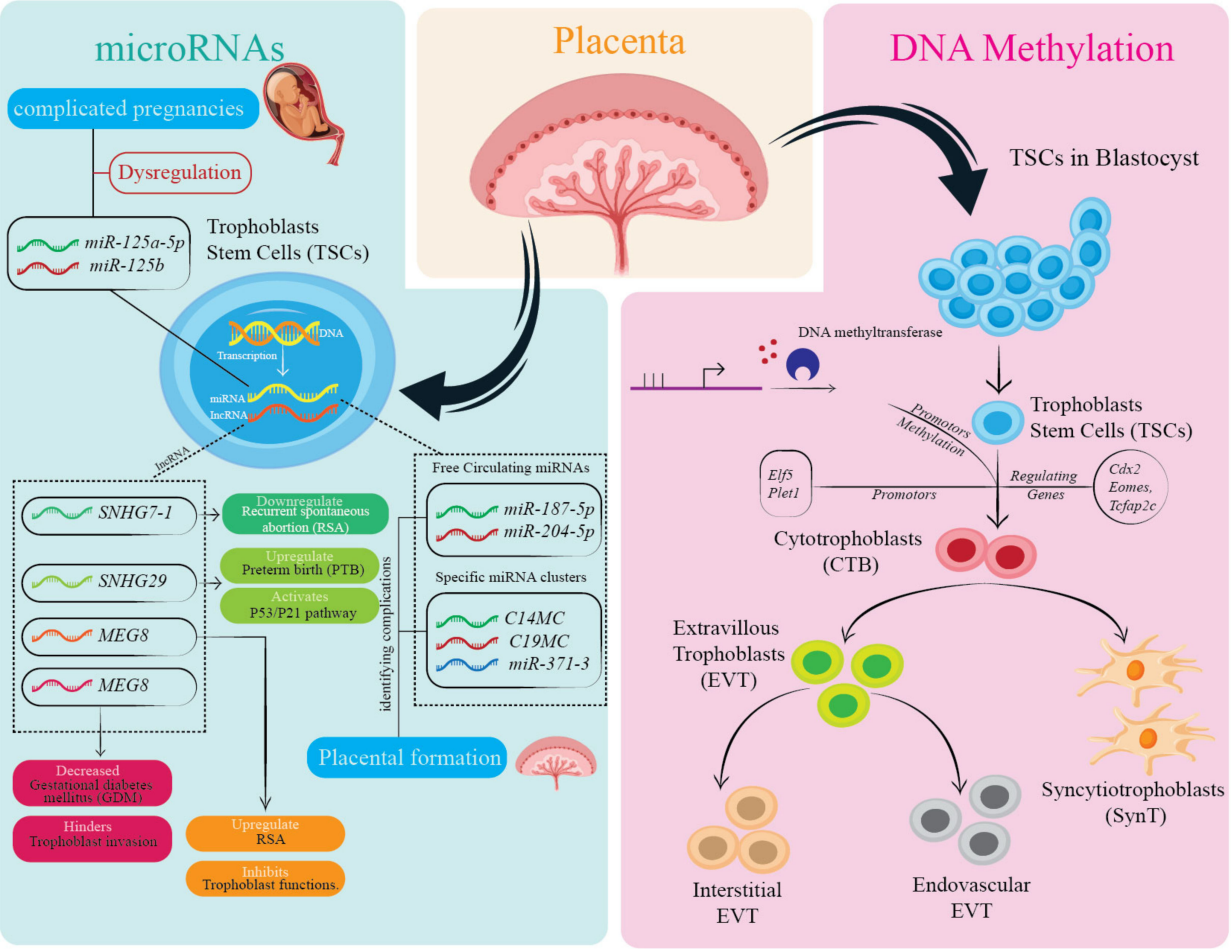
Overview of epigenetic and molecular regulation of trophoblast cell function and differentiation. This figure highlights the influence of *microRNAs* (e.g., miR-125a, miR-182-5p) and DNA methylation on TSCs and their differentiation into various trophoblast cell types, including CTBs and EVTs. Dysregulation of these factors is associated with pregnancy complications. Specific genes and proteins play critical roles in placental formation and trophoblast function. The cellular pathways and components illustrated contribute to maintaining placental health, with disruptions linked to adverse pregnancy outcomes.

Collectively, these transcription factors develop a complex regulatory network that maintains trophoblast progenitor populations and facilitates lineage differentiation. Although numerous elements of trophoblast regulation are reserved in murine and human models, remarkable differences persist, especially concerning regulating *CDX2* expression in early human development. More research is required to comprehensively clarify the interactions of these factors in regulating human trophoblast differentiation. **Figure 4** provides a visual summary of the different mechanisms impacting trophoblast differentiation and function.

## 4 Clinical implications

### 4.1 The link between trophoblast pathways dysregulation and pregnancy complications

The placenta is pivotal for supporting pregnancy and affecting maternal and fetal health. There is a strong correlation between abnormal placenta development and various pregnancy disorders, including hypertensive disorders of pregnancy (HDP), unexplained stillbirth, IUGR, miscarriage, and GDM, which are responsible for a significant amount of illness and death in both mothers and newborns (126). PE is one of the primary types of the HDP (110). Significantly, PE is the most concerning pregnancy disorder; it is characterized by the development of high blood pressure, proteinuria, and dysfunction of organs, which can occur after 20 weeks of pregnancy (140). It is also characterized by the sudden occurrence of a widespread seizure (107).

Alterations in the expression of genes and epigenetic modifications of trophoblast’s different signaling components are linked to various gestational disorders. Additionally, the placenta of complete hydatidiform mole (CHM) has a more significant number of β-catenin-positive EVT nuclei than normal tissues (141). This suggests that abnormal invasion in this pregnancy disorder may be caused by aberrant Wnt signaling.

The genes *APC* and *sFRP2* increase methylation in choriocarcinoma cells, suggesting that the inactivation of negative regulators of Wnt signaling likely plays a role in the development and advancement of trophoblastic cancer cells (142).

Cytomegalovirus infection, which is believed to be a potential cause of spontaneous abortion and preterm delivery, has been found to reduce the growth and spread of trophoblast cells via inhibiting Wnt signaling. The virus caused changes in the distribution of β-catenin. It led to its breakdown and decreased the activity of a standard Wnt reporter when trophoblastic cells were infected (143).

The dysregulated VEGF pathway significantly takes part in placental dysfunction. In PE, serum and placental levels of *sFlt-1* are upregulated, and *VEGF* and *PlGF* are downregulated (144). Also, *VEGFR-1* and *VEGFR-2* are overexpressed in the placenta, leading to placental dysfunction through endothelial dysfunction, hypertension, and proteinuria (145). Since elevated sFlt-1 levels may decrease VEGF and PlGF concentrations, it may be a critical factor in these VEGF system changes.

Around 15% of pregnancies end in miscarriage. Many early miscarriages are caused by trophoblastic lineage issues and chromosomal abnormalities that impair placentation (146). It was reported that women with idiopathic recurrent pregnancy loss have placenta with impaired CTB/STB bilayer structure and trophoblastic column formation (129). The YAP/TEAD4 signaling pathway, critical for CTB proliferation, was dysregulated in miscarriage tissues, highlighting the importance of transcriptional regulation in the trophoblast role (147).

### 4.2 Exploring the potential therapeutic strategies to address abnormal trophoblast function and improve pregnancy outcomes

Considering the vital role of trophoblast cells in placental function, therapeutic approaches restoring their optimal role may be promising in addressing pregnancy complications. EVT invasion, vascular remodeling, and immune tolerance are critical processes impaired in PE, IUGR, and GDM (148). Extensive previous research has successfully identified and described the role of CTBs, EVTs, and STBs in various pregnancy complications. Abnormal placentation commonly occurs due to the absence or incomplete alteration of the spiral arteries by EVTs (149).

Fetal growth restriction (FGR) refers to a condition in which the fetus exceeds its genetic growth capacity and is often accompanied by abnormalities in the morphology and function of the placental STB (150). The administration of an IGF1 plasmid directly to the placenta in a mouse model of FGR helps sustain fetal growth after surgical induction. This approach leads to enhanced IGF1 expression in the placenta, which increases the expression of glucose and amino acid transporters. Thus, it is recommended that IGF modulation be explored as a possible therapeutic approach (151).

Placental hypoxia is believed to result in STB stress, leading to a decrease in nutrient transportation to the fetus. This condition also produces antiangiogenic substances, such as soluble sFLT1, a PE biomarker (152). Several investigations were conducted using siRNA against sFLT1 during pregnancy, yielding encouraging outcomes. One of the stress response proteins, Heme oxygenase 1 (HO-1), is a potential target for delivering genes to treat PE. HO-1 is involved in angiogenesis and helps to suppress the *sFLT1* expression (153). HO-1 mRNA levels are lower in PE compared to standard pregnancy cases, which could be the reason for the higher levels of circulating sFLT1. The impact of the HO-1 upregulation has been investigated utilizing mesenchymal stem cells (MSC).

Trophoblastic adrenomedullin (ADM) represents a possible target for early-onset PE. ADM controls trophoblast differentiation and function, and its downregulation has been connected to abnormal placentation (154). The faulty expression of trophoblastic ADM is a fetal factor contributing to early-onset PE by impacting the distinguishing and functioning of trophoblasts (155), leading to disturbances in the usual placenta growth (156). These findings emphasize the potential clinical application of ADM in predicting early-onset PE and suggest that delivering ADM directly to the placenta could be a viable treatment option.

## 5 Current challenges and future directions

### 5.1 Organoids of trophoblasts

Organoid technology serves as an effective method for simulating mimics of human organ physiological and pathological development. Organoids enable new regenerative medicine research by incorporating gene-editing technology. Over the last several years, significant advancements have been made in the study of trophoblast organoids, assisted by 3D culture technology and functional cells derived from stem cells (157). These organoids have effectively aided placental villous trophoblast formation and communication, enabling a more thorough examination of early placental development. Multiple publications on first-trimester placental villi support primary placental cell-based organoids, which meet all criteria for trophoblast cells, including gene expression and similarity to human primary placenta cells (106).

Placental organoids can also be used in clinical research to study fetal infection in placental infectious diseases and monogenic hereditary diseases through gene editing, screening, and toxicological analysis (158). The current model has restrictions, and further studies are needed to improve our understanding of placentation.

### 5.2 Single-cell RNA sequencing

Single-cell RNA sequencing (sc-RNAseq) technology has significantly enhanced the comprehension of placental development. sc-RNAseq facilitates investigating gene expression at the individual cell level, unveiling vital information concerning the cell subpopulations that exist through different stages of gestation (159, 160). Studies utilized an artificially grown human embryo in a laboratory setting to determine when the trophoblast lineage is established and the necessary regulatory function of the T-box transcriptional factor 3 in the initial formation of STBs (160). Between 6 and 14 weeks of early gestation, researchers analyzed different types of placental and decidual cells, created a transcriptomics atlas, identified cell type-specific transcription factors, and studied significant ligand-receptor interactions. These findings elevated our understanding of placental development by highlighting the crucial regulatory mechanisms involved in early placental development (161).

### 5.3 Placenta-on-chip

The placenta-on-chip model is considered an innovative microfluidic platform with distinct physical properties that enable accurate manipulation of cell arrangement and subcellular environments, providing a fundamental framework and mechanism for the organ-on-chip model (162). Several researchers have attempted to develop a system of placental microchips that can replicate the process of material exchange in the body to investigate element transport and metabolism and prevent infections in the placental barrier (163).

The placenta-on-a-chip device accurately represents the *in vivo* environment by displaying a remarkable abundance and compactness of microvilli on the surface of trophoblastic cells. Thus, this enables the examination of intercellular communication and its influence on placental function, significantly enhancing our comprehension of the relationships between the structure and function of organs (162). Nevertheless, enhancements are required in domains including regulating liquid flow and monitoring technology encompassing a broader spectrum of cells and microorganisms to attain advanced technology and widespread cost-effective implementation.

## 6 Conclusion

This review elucidates the intricate network of molecular pathways that regulate trophoblast migration, differentiation, and overall placental development, highlighting their critical roles in ensuring successful pregnancy outcomes. The Wnt, Notch, TGF-β, and VEGF signaling pathways are central to regulating these processes, with their dysregulation being strongly associated with pregnancy complications such as PE, IUGR, and recurrent miscarriage. By understanding these pathways and the epigenetic and post-transcriptional mechanisms that modulate them, we can gain valuable insights into the pathogenesis of placental disorders.

Furthermore, emerging technologies like trophoblast organoids and placenta-on-chip models improve placental biology knowledge. These models replicate crucial placental development and help study pregnancy disorder mechanisms. The ability to reproduce trophoblast functions in a controlled setting opens new avenues for drug testing and molecular mechanisms of disease. Future research must translate molecular insights into clinical applications to improve maternal and fetal health.
